# Vaccination Schedule under Conditions of Limited Vaccine Production Rate

**DOI:** 10.3390/vaccines10010116

**Published:** 2022-01-13

**Authors:** Roger Książek, Radosław Kapłan, Katarzyna Gdowska, Piotr Łebkowski

**Affiliations:** Faculty of Management, AGH University of Science and Technology, 30-059 Cracow, Poland; rksiazek@zarz.agh.edu.pl (R.K.); rkaplan@zarz.agh.edu.pl (R.K.); plebkows@zarz.agh.edu.pl (P.Ł.)

**Keywords:** COVID-19 vaccination, vaccination schedule, optimization, scheduling, mathematical model, decision-making, herd immune

## Abstract

The paper is devoted to optimal vaccination scheduling during a pandemic to minimize the probability of infection. The recent COVID-19 pandemic showed that the international community is not properly prepared to manage a crisis of this scale. Just after the vaccines had been approved by medical agencies, the policymakers needed to decide on the distribution strategy. To successfully fight the pandemic, the key is to find the equilibrium between the vaccine distribution schedule and the available supplies caused by limited production capacity. This is why society needs to be divided into stratified groups whose access to vaccines is prioritized. Herein, we present the problem of distributing protective actions (i.e., vaccines) and formulate two mixed-integer programs to solve it. The problem of distributing protective actions (PDPA) aims at finding an optimal schedule for a given set of social groups with a constant probability of infection. The problem of distributing protective actions with a herd immunity threshold (PDPAHIT) also includes a variable probability of infection, i.e., the situation when herd immunity is obtained. The results of computational experiments are reported and the potential of the models is illustrated with examples.

## 1. Introduction and Background

The COVID-19 pandemic, which has been ongoing since 2020, has exposed many problems related to the management of efforts to combat and counter global threats. One problem is the management of mass vaccination campaigns, including the creation of transparent mechanisms for vaccine distribution during a pandemic based on clear rules and legitimate criteria for product dispensing. For pandemic containment, this issue is critical in the initial period after the vaccine is accepted for widespread use when preparation supplies are still insufficient for the entire population.

The problem with limited access to vaccines during the initial phase becomes a logistical and ethical problem [1,2,3]. As noted by Bolcato et al. [4], it is necessary to define criteria for dividing the population into groups (by, for example, age, profession, and immunity) and to determine their hierarchy. The topic of rational vaccination scheduling was addressed by Hughes et al. [2]. Yang et al. [5] created the following hierarchy of vaccine distribution for the COVID-19 pandemic in China based on the criterion of occupational and social groups: the first phase of vaccination should cover people performing jobs critical to the functioning of basic services; that is, essential workers including staff in healthcare, law enforcement, security, nursing homes, social welfare institutes, community services, the energy sector, the food industry, the transportation sector, and overseas workers/students (the size of this group is estimated at 49.7 million people); the second phase should include older adults, individuals with underlying health conditions and pregnant women (another 63.6 million people) as this would reduce the number of patients with severe COVID-19 outcomes requiring hospitalization, critical care admission, and the number of deaths; in the third phase adults without underlying health conditions and children (784.8 million people) would be vaccinated, as this would reduce the number of severe and mild cases, and reduce virus transmission.

Factors related to the mechanism and dynamics of a specific epidemic and the social reception of mass vaccination campaigns should also be considered when developing a vaccination strategy. Piraveenan et al. [6] and Savoia et al. [7] highlight the key drivers that may influence vaccine uptake: the epidemic potential of the pathogen, localized transmission potential of the pathogen, safety concerns with the vaccine, strength, and flexibility of public health delivery systems, public investment in resources for immunization, and local ownership, individual normative behaviors, the level of vaccine hesitancy by the population, and local context and its multi factorial determinants. The results of studies conducted in China [8] and the USA [9] during the COVID-19 pandemic show that the level of vaccine hesitancy by the population is an important factor for the efficiency of vaccination campaigns, especially when vaccination is free and non-mandatory.

In this paper, the authors focus exclusively on the logistical-organizational (rather than moral) aspect of vaccine distribution during the initial phase with limited access to the product, i.e., planning the distribution of available vaccines among social groups defined based on specific criteria. The selection of criteria for dividing the population into groups and the methods of group stratification is not the subject of our study. The presented approach to planning mass vaccination campaigns assumes that the division of the population into groups with an assumed (accurately defined) probability of infection has already been made, and we do not discuss the key for this division. The division can be based on population density, occupation, or age. The problem of distributing protective measures with a threshold of herd immunity was formulated to optimize the use of vaccines and thus minimize the incidence of the disease.

The main contribution of this paper is to formulate the problem of distributing protective actions (i.e., vaccines) with a herd immunity threshold aiming at maximizing the herd immunity in the situation of limited availability of resources. We present two mixed integer programming (MIP) models for the scheduling vaccination problem:

- the problem of distributing protective actions (PDPA)–(pessimistic), which assumes high availability of vaccines and a constant probability of disease in each social and occupational group that does not decrease even if the number of vaccinated people increases;
- the problem of distributing protective actions with a herd immunity threshold(PDPAHIT)–(optimistic) which assumes high availability of vaccines and a variable probability of disease in each social and occupational group that changes as the threshold of herd immunity is reached.

These models should be used to help solve the problem of vaccine distribution among broadly defined recipients, such as regions of a country, members of a federation, or community groups. Furthermore, the construction of the models allows them to be used hierarchically, from national structures to small distribution units.

The remainder of this paper is structured as follows: Section 2 presents the methodology based on mixed-integer programming (MIP). The newly-developed MIP models for the problem of distributing protective actions and the problem of distributing protective actions with a herd immunity threshold are presented. Section 3 describes the computational experiments, while Section 4 reports the results and their interpretation in terms of their usefulness for decision-making units (DMU). To test, verify, and validate the model, publicly available data published in [10,11,12,13,14] were used. In Section 5, we concluded and made recommendations for further research.

### Positioning of the Paper

The global experience of the SARS-CoV-2 pandemic and the widespread introduction of mass vaccination in 2021 as a countermeasure against its spread has necessitated more research on strategies for building natural and vaccine-induced immunity and managing population vaccination campaigns [15,16,17,18,19].

In June 2020, the World Health Organization (WHO) issued recommendations [20] on priorities to be applied when organizing vaccination campaigns. They are the basis for overall planning and implementation of activities in individual countries, e.g., [21]. The prices and methods of vaccine distribution that differ between countries depending on their affluence have become an important issue [22,23]. Vaccination strategies are analyzed from multiple global and local perspectives and concerning various parameters such as population specifics, vaccine availability, vaccination logistics, etc. The goal of this research is to develop an optimal vaccination scenario that would reduce the rate of infection and the level of mortality due to COVID-19 in a given area. A compartmental predictive model of pandemic development that took vaccination into account was presented by [24]; they divided population N(t) into the following compartments: susceptible S(t), exposed S(t), symptomatic infectious $I_{S}\left(t\right)$, asymptomatic infectious $I_{A}\left(t\right)$, recovered R(t), dead D(t) and vaccinated V(t). Research using models of this type was also presented in [25,26,27,28]. The models presented in [29,30] consider the effect induced by vaccination and continuous use of non-pharmaceutical interventions (NPI). Bubar et al. [31] conducted a study using an age-stratified SEIR model to identify the age groups that should receive the highest priority in access to vaccination. Computational experiments on data corresponding to populations with the age distributions and modeled contact structures of the United States, Belgium, Brazil, China, India, Poland, South Africa, and Spain showed that the greatest reduction in infectiousness is achieved when the 20+ age group is vaccinated, and the greatest reduction in mortality is the result of administering the vaccine to the 60+ group first. Campos et al. [32] used data from Brazil and the SIR model, Foy et al. [33] employed the SEIR model and data from India, and Zhao and Chen [34] utilized the SEIAR model and data from Wuhan in China, and they all drew similar conclusions. The classical parametric deterministic mathematical models of infectious diseases (e.g., SIR, SEIR, and SIDR) have proven useful for predicting pandemic development, but require too many assumptions to be used with good effect in evaluating the outcomes of specific intervention strategies undertaken to counter the spread of the virus [35,36,37,38].

Wrigley-Field et al. [39] analyzed vaccination strategies based on COVID-19 mortality data in the U.S. and found a correlation with both age groups and the ethnic structure of geographic regions. They showed that vaccination strategies based on geographic criteria were more equitable and effective (i.e., prevented more deaths) than strategies based on age. Fuady et al. [27] highlighted the need to study the effectiveness of vaccination campaigns with different criteria for dividing the population into hierarchical groups. Using the SIQDR model, they made predictions of COVID-19 pandemic development in Indonesia for several scenarios of delivering vaccines: without vaccination, fair distribution, and targeted distribution to five and eight districts with the highest COVID-19 incidence in West Java. The results showed that over 33 months of viral spread, the most effective reduction in the number of active cases was achieved when vaccines were distributed according to a fair distribution scenario. Similar research was run on data collected in New York City by Chen et al. [40]; they showed that dynamic myopic policies were the most effective for the population studied and the adopted distribution criterion. Looking at the results from an ethical perspective, they argued that high efficiency in terms of the confirmed cases and deaths can be achieved by sacrificing a small portion of equity. Therefore, local demographic structure, intersectional risks, and large-scale and small-scale geographic stratification should be considered when optimizing vaccination strategies. The population can be prioritized on more than one factor e.g., key occupations and age [5]. Apart from medics, truck drivers were listed as crucial occupations [41]. Yu et al. [42] have shown that dynamic vaccination strategies, where it is acceptable to redirect vaccines to regions with the temporarily highest demand, are most effective. Moreover, to provide the most effective protection, the criterion for stratifying the population may change depending on the number of available vaccines, e.g., with low vaccine availability, the prioritization of the population may be related to occupational groups, but with a high number of vaccines, it may be related to age groups [43]. Depending on the availability of vaccines, the highest effectiveness may result from giving top priority to different age groups [44].

An essential aspect in planning a mass vaccination campaign in a pandemic is the public attitude resulting from the way the country organized the immunizations against infectious diseases in the past, the percentage of vaccinated people [7,45], as well as the beliefs about the efficacy and possible risks associated with the vaccine against the virus of the current epidemic (in this case, the COVID-19 pandemic). Based on data from China, Wang et al. [8], noted that vaccine price and vaccination convenience as well as general health education and communication from authoritative sources may well be factors limiting the propensity to undergo vaccination. Information about vaccination and particular vaccines shared on social media [9] is equally important for building public awareness and beliefs.

Another issue is the management and implementation of the vaccination procedure, which is also the subject of scientific research (e.g., [46,47]), but falls beyond the scope of this article, as do the issues of vaccine production and supply chain organization [48,49,50,51,52,53]. Given the prediction of future pandemics [54], further exploration of this topic is warranted.

## 2. Materials and Methods

As discussed in Section 1, the problem of distributing a limited number of vaccines during an ongoing pandemic is very complex. Three contributing groups of factors include: (I) different versions of the vaccine characterized by parameters such as (1) availability, (2) effectiveness in preventing infection, (3) reduced risk of severe disease, (4) number of doses required, (5) shelf-life, (6) requirements that must be met during vaccine transport; (II) the characteristics of a given pandemic described by parameters such as (1) effects on the human organism, which may depend on age, sex, (2) pre-existing diseases, etc., (3) transmission and conditions of pathogen dissemination; (III) the characteristics of a region described by parameters such as (1) population density, (2) the dominant economic sector in the region.

It should be noted that the main goal of starting a vaccination program is to prevent fatal infections, and this goal can be realized on two levels:

1. Direct level—lowering the probability of severe disease for a vaccinated individual. All vaccinated individuals reduce the overall probability of fatal cases in the region.
2. Indirect level—lowering the probability of infection for vaccinated individuals. The vaccine minimizes the probability of infection so that a growing group of vaccinated individuals lowers the probability of infection.

In addition, minimizing the probability of severe disease does not depend only on the probability of infection in a given region or social group but also on the individual’s susceptibility to a severe course of the disease. For example, the main factors underlying severe COVID-19 infection are comorbidities and age (see Table 1) [10].

According to the analyses conducted by the Centers for Disease Control and Prevention (CDC), the probability of infection is practically independent of age, which cannot be said about the probability of hospitalization and potential death. As shown in Table 1, in both cases, there is a strong correlation between these parameters and the age of an individual. On the other hand, in [55] a correlation between the probability of infection and population density was shown. This dependence is also discussed in the study [56].

In summary, the probability of a severe course of the disease in a vaccinated person can be illustrated by the following equations:

(1) $$P_{S_{zg}}=P_{S_{zk}}P_{zg}\left(1-S_{zg}\right)$$

(2) $$P_{S_{zk}}=P_{zk}\left(1-S_{zk}\right)$$

(3) $$P_{zk}=\left(1-Z\right)P_{z}+ZP_{z}\left(1-S_{zk}\right)$$

where:

$P_{S_{zg}}$— the probability of severe course of the disease,
$P_{S_{zk}}$— the probability of infection in the vaccinated individual,
$S_{zg}$—the effectiveness of the vaccine against the severe course of the disease,
$S_{zk}$—the effectiveness of the vaccine against the infection,
$P_{zg}$—the probability of death of a given individual, e.g., depending on the age,
$P_{zk}$—the probability of an infection in a given area during an active vaccination program,
*Z*—the percentage of vaccinated individuals expressed as a fraction of the total population,
$P_{z}$—the probability of infection in a given area, e.g., depending on the level of urbanization.

Of course, the above relationships are simplified and, as such, do not take into account many issues such as variation in vaccine efficacy depending on the number of doses received or the vaccine manufacturer. For example, for the COVID-19 pandemic, these factors are presented in Table 2 [11,12,13,14].

There are four available vaccines. To achieve the declared efficacy, three vaccines need to be administered in two doses. In the case of Johnson and Johnson, a single dose is sufficient. The declared effectiveness against COVID-19 infection ranges from 67% to 95%. For two-dose vaccines, efficacy against the severe course of COVID-19 is close to 100%, and for the single-dose vaccine, it is 85% (see also [57,58]).

Of the parameters listed above, the percentage of vaccinated people appears to be the decisive factor. The responsibility for determining what percentage of a given population group in a given territory will be vaccinated rests with the national or regional governments. It is important to note that it is within the power of policymakers to reduce the likelihood of infection in a given area by implementing non-pharmaceutical interventions (NPIs). The impact of these interventions is not the focus of this study, yet the model itself (as presented below) makes it possible to account for NPIs’ impact resulting from their effect on the probability of infection in a given group. The remaining parameters are specific to a given region and the characteristics of the infectious disease and, as such, are independent of policymakers.

The percentage of vaccinated individuals in a given social group depends on many variables, including socio-cultural factors. However, as we learned during the COVID-19 pandemic, the main problem in the initial phase of the vaccination program is the limited amount of products.

In this work, an optimization problem of determining optimal vaccination schedules is formulated; it aims at finding such a vaccination schedule that minimizes the probability of infection. The distribution of vaccines among different population groups, the scheduling of vaccination in these groups, the allocation of individuals to specific vaccination points, and ensuring appropriate transportation of vaccines is extremely difficult, especially with limited resources (limited number of vaccines, vaccination points, and means of transportation) and in the face of the necessity to begin a vaccination program immediately.

However, this problem has been researched for many years, and science has already found actionable solutions. The approach adopted in this paper is generally referred to as production planning. There is a close similarity between production planning in a company or region and scheduling vaccination in society. The difference comes down to the object on which technological procedures are performed and thus to the different formulation of the optimization objectives of production processes and vaccination processes. In production scheduling, we deal with parts, equipment, or a product of the human mind. Therefore, delays in the implementation of production tasks are acceptable. In the vaccination process, especially during a pandemic, any delay can result in severe disease and the death of a patient.

A proven tool used in production planning (and thus in vaccination planning) is mathematical programming, especially linear programming. Linear programming requires precise identification of acceptable solutions to a modeled problem and the best solution given the adopted optimization criterion (criteria). Although all constraints and the objective function in linear programming must be linear and the feasible region must be convex, there are excellent algorithms for solving nonlinear problems: integer and binary. In the case of scheduling vaccinations, we are dealing with integer programming, e.g., we vaccinate a certain integer (rather than a fractional—number of people) and binary programming (e.g., we provide a given group of people with vaccines or we do not provide vaccines). The branch-and-bound algorithm and the branch-and-cut method allow finding optimal solutions for the assumed criterion. Optimal means the best among all possible solutions. The above-mentioned methods are very successful and efficient for solving mixed-integer programming problems and providing optimal solutions [59,60,61].

### 2.1. The Problem of Distributing Protective Actions on the Example of Vaccination

The problem of distributing protective actions (PDPA) is formulated as follows. Over an assumed planning horizon *T* (e.g., several consecutive weeks t∈T), a community (e.g., citizens of a country) should be subject to available protective actions *V* (e.g., vaccination using several different preparations) to minimize the effects of an adverse phenomenon (e.g., in the case of a pandemic, such an effect is the number of people infected with the virus or the number of deaths). The population is divided into $\bar{R}$ homogeneous groups, e.g., by age, occupation, or place of residence. However, the method and criteria for division are beyond the scope of this decision problem. The size of each group r∈R ($d_{r}$), as well as the probability of an adverse phenomenon for an individual in group *r* if no protective action is taken ($p_{r}^{R}$) are known.

For each protective action v∈V, e.g., vaccination with a particular preparation, the probability of an adverse phenomenon for an individual receiving it is known ($p_{v}^{V}$). In the initial period after the introduction of a protective measure (e.g., after vaccine approval), the demand is high, while availability may be constrained due to the limited production capacity and distribution points (e.g., vaccination centers). Thus, it is possible to determine the maximum available number of units of each protective action *v* in period t∈T ($b_{vt}$), e.g., the number of vaccinations with a given preparation, and the maximum available number of all protective actions available in period *t* ($C_{t}$), e.g., the total number of doses of all available vaccines. It is necessary to determine how many times a protective measure *v* should be applied in a social group *r* in period *t* to minimize the effects of an adverse phenomenon in the entire community over the assumed planning horizon. The notation used is presented in Table 3 and the associated mixed-integer program is written as model (4)–(10).

(4) $$\min:\sum_{r\in R}\sum_{t\in T}I_{rt}$$

(5) $$\left(d_{r}-\sum_{v\in V}x_{rv0}\cdot p_{v}^{V}\right)\cdot p_{r}^{R}=I_{r0},\;r\in R;$$

(6) $$\left[\begin{array}{c} p_{r}^{R}\cdot \left(d_{r}-\sum_{v\in V}\sum_{t^{\prime }\in T:t^{\prime }<t}x_{rvt^{\prime }}-\sum_{t^{\prime }\in T:t^{\prime }<t}I_{rt^{\prime }}\right)+\\ +p_{r}^{R}\cdot p_{v}^{V}\sum_{v\in V}\cdot x_{rvt} \end{array}\right]=I_{rt},\;r\in R,t\in T:t>0;$$

(7) $$\sum_{r\in R}x_{vrt}\leq b_{vt},\;v\in V,t\in T;$$

(8) $$\sum_{v\in V}\sum_{t\in T}x_{vrt}\leq d_{r},\;r\in R;$$

(9) $$\sum_{v\in V}\sum_{r\in R}x_{vrt}\leq C_{t},\;t\in T;$$

(10) $$I_{rt}\geq 0\;r\in R,t\in T;$$

(11) $$x_{rvt}\geq 0,\;r\in R,v\in V,t\in T;$$

The objective function (4) ensures that the impact of the adverse phenomenon is minimized across the community over the considered period. It is achieved by minimizing the expected value of the number of people subject to the effects of the adverse phenomenon in all social groups over the entire planning horizon. Constraints (5) and (6) are used to determine the expected value of the number of people subject to the adverse phenomenon at each period in each group. In each social group r∈R, the expected value of the number of individuals subject to the adverse phenomenon in period t=0 is calculated for that part of the group $(d_{r}-\sum_{v\in V}x_{rv0}$)that has not been subjected to protective actions (constraint (5)); the value of the variable $x_{rv0}$ should be equal to 0 in each group for each protective action when the distribution of these measures is at the initial stage. When the planning horizon covers any period, the value of the variable $x_{rv0}$ should be greater than 0 and should result from the protective action application records. According to the formula used in constraint (6), the expected value of the number of individuals belonging to group *r* subject to the adverse phenomenon in period *t* depends on the probability of its occurrence in individuals not subjected to protective actions in this period and earlier periods, as well as on the probability that expresses the effectiveness of protective actions in individuals subjected to those actions in the considered period *t*. Constraints (7)–(9) ensure that the number of protective action applications does not exceed the available limits in particular periods, and the number of doses allocated to each social group r∈R does not exceed its size. It should be emphasized here that parameter $C_{t}$ represents the system capacity in period t, e.g., the maximum number of doses of all the available vaccines to be provided to the society in period *t*, while parameter $b_{vt}$ is the number of doses of every vaccine *v* available in period *t*, so the relationship between $C_{t}$ and $\sum_{v\in V}b_{vt}$ is obvious, but it may not be necessarily $C_{t}=\sum_{v\in V}b_{vt}$. It is possible that in planning period *t* the total number of doses of all the available vaccines (i.e., $sum_{v\in V}b_{vt}$) can be greater than the system’s capacity to provide them to the public (so $C_{t}$ vaccinations are done and some number of doses remains unused). Moreover, the opposite situation is possible as well, if the total number of doses of all the available vaccines (i.e., $\sum_{v\in V}b_{vt}$) is less than $C_{t}$, the number of provided protective actions is equal to $\sum_{v\in V}b_{vt}$) (i.e., all the available doses are used and some vaccination points must be idle for some time because of the insufficient number of available protective actions). We use these two parameters in the model, because we would like it to be a decision support tool that does not require in every planning period *t* a pre-analysis of the relationship between $C_{t}$ and $\sum_{v\in V}b_{vt}$, but can find the optimal solution for every data instance. In turn, constraints (10) and (11) determine the format of the decision variables and ensure the integrity of the model.

### 2.2. The Problem of Distributing Protective Actions with a Herd Immunity Threshold

The problem of distributing protective actions with a herd immunity threshold (PDPAHIT) is an extension of the general problem of distributing protective actions (PDPA) presented in Section 2.1. Analogously to the previous problem, the goal is to minimize the effects of an adverse phenomenon (e.g., the number of infections or deaths) over an assumed planning horizon *T* in a population *R* divided into $\bar{R}$ homogeneous groups by applying available protective action v∈V. An important modification of the problem is to consider the threshold for achieving herd immunity to the considered adverse phenomenon, i.e., the proportion of individuals subjected to protective actions that ensure the herd immunity in the group (*f*). In this problem, the herd immunity threshold for the whole population is assumed to be the same as for each separated group r∈R. The fact that the herd immunity threshold has been reached is represented by the binary variable $y_{rt}$, which takes the value equal to 1 if group *r* in period *t* has reached herd immunity. We assume that once collective resilience is achieved in group *r* in period *t*, it persists for subsequent periods until the end of the planning horizon. As in the previous task, the quantities represented by the parameters $d_{r}$, $p_{r}^{R}$, $p_{v}^{V}$, $b_{vt}$, and $C_{t}$ are known. It is necessary to determine how many times a protective action *v* should be applied in a social group *r* in period *t* to minimize the effects of an adverse phenomenon in the entire community over the assumed planning horizon. The notation used is presented in Table 3 and the associated mixed-integer program is written as the model (12)–(20).

(12) $$\min:\sum_{r\in R}\sum_{t\in T}I_{rt}$$

(13) $$\left(d_{r}-\sum_{v\in V}x_{rv0}\cdot p_{v}^{V}\right)\cdot p_{r}^{R}=I_{r0}+M\cdot y_{r0},\;r\in R;$$

(14) $$\left[\begin{array}{c} p_{r}^{R}\cdot \left(d_{r}-\sum_{v\in V}\sum_{t^{\prime }\in T:t^{\prime }<t}x_{rvt^{\prime }}-\sum_{t^{\prime }\in T:t^{\prime }<t}I_{rt^{\prime }}\right)+\\ +p_{r}^{R}\cdot p_{v}^{V}\sum_{v\in V}\cdot x_{rvt} \end{array}\right]=I_{rt}+M\cdot y_{rt},\;r\in R,t\in T:t>0;$$

(15) $$\sum_{r\in R}x_{vrt}\leq b_{vt},\;v\in V,t\in T;$$

(16) $$\sum_{v\in V}\sum_{t\in T}x_{vrt}\leq d_{r},\;r\in R;$$

(17) $$\sum_{v\in V}\sum_{r\in R}x_{vrt}\leq C_{t},\;t\in T;$$

(18) $$\sum_{v\in V}\sum_{t^{\prime }\in T:t^{\prime }\leq t}x_{rvt^{\prime }}\geq f\cdot d_{r}\cdot y_{rt},\;r\in R,t\in T;$$

(19) $$y_{rt}\geq y_{r,t-1},\;r\in R,t\in T:t>0;$$

(20) $$x_{rvt}\geq 0,\;r\in R,v\in V,t\in T;$$

(21) $$I_{rt}\geq 0\;r\in R,t\in T;$$

Model (12)–(20) is an extension of model (4)–(10). The objective function (12) is identical as in PDPA model (i.e., objective function (4)). Constraints (13) and (14) are used to determine the expected value of the number of individuals subject to the adverse phenomenon at each period in each group, but each has been modified with components $M\cdot y_{r0}$ and $M\cdot y_{rt}$ respectively added to the equation on the right side. This ensures that the achievement of the herd immunity threshold (then $y_{rt}=1$) is taken into account when determining the expected number of individuals in group *r* subject to an adverse phenomenon in period *t*. From the point of view of model performance, when for group *r* in period *t*$y_{rt}=1$, then the value of the whole component $M\cdot y_{rt}=M$, so for the sum $I_{rt}+M\cdot y_{r0}$ the value of $I_{rt}$ will be of negligible importance. The objective function (12), which minimizes the sum of $I_{rt}$ variables, will necessitate that for considered *r* and *t*$I_{rt}$ will take the value 0, which can be interpreted as the elimination of the threat resulting from the effect of the adverse phenomenon, i.e., achieving herd immunity in period *t* in group *r*. The fact that herd immunity is achieved in group *r* in period *t*, represented by $y_{rt}=1$, is closely related to the number of individuals in this group who have been subjected to protective actions in periods before *t*. This relationship is reflected in constraint (18), which guarantees that $y_{rt}=1$ in group *r* in period *t*, if the total number of individuals in this group subjected to protective actions in periods before *t* (left side of the inequality) is not less than the required proportion of protective measure applications to achieve collective immunity *f* (right side of the inequality). Constraint (19), on the other hand, ensures that once herd immunity is achieved in group *r* in period *t*, it persists for subsequent periods until the end of the planning horizon. Constraints (15)–(17) are identical to constraints (7)–(9) and ensure that the number of applications of protective actions does not exceed the available limits in both periods, while the number of doses allocated to each group r∈R does not exceed its size. The format of the decision variables and the integrity of the model are ensured by constraints (20) and (21).

## 3. Results

The example analysis was conducted for the territory of a European country; however, to present all functionalities of the model, it was necessary to make assumptions on the differences between particular vaccines and the probability of infection of particular social groups. It should also be noted that the proposed model is a simplification of dependencies (I)–(III) presented in Section 2, which is why it aggregates individual probabilities.

The assumptions related to social groups, vaccine availability, and vaccine delivery schedules:

**Social groups**—the first issue is to identify social groups in a given territory with a constant probability of infection. In the presented example, the territorial division of the country was used, and the probability of infection was related to the population density, which is presented in Table 4.

Table 4 shows the population of a territory and the constant probability of infection. An exemplary division into social groups could be as follows: paramedics, general practitioners, police officers, firefighters, etc.

**Available vaccines**—these are characterized only by the probability of preventing infection. This data for individual vaccines are presented in Table 5.

The data presented in Table 5 are a simplification of the problem described in Section 2 and apply only to the probability of infection.

**Vaccine delivery schedule**—the planned delivery schedule for each vaccine type is shown in Table 6.

We will use two districts to discuss the results:

- “District 1” has a medium-sized population (2,901,225 residents) and one of the lowest probabilities of infection (0.01);
- “District 10” has a relatively small population (1,181,533.00 inhabitants) and one of the highest probabilities of infection (0.09).

The results for “District 10” are shown in Table 7 which includes the number of people vaccinated with each type of vaccine in the consecutive analyzed periods. In contrast, for “District 1”, the model did not allocate vaccines in any analyzed cycle.

As we can see from the example of the analyzed districts, in PDPA the distribution started from the areas with the highest probability of infection and large populations. At the same time, the pandemic was allowed to develop in districts with smaller populations (“District 1”). Such a procedure results from the fact that, for example, “District 10”, due to its size, was threatened by much higher dynamics of pandemic development. Only after stabilizing the situation in districts of this type did the vaccination program begin in subsequent locations with smaller populations and a lower probability of infection for which the dynamics of pandemic development present the greatest risk in a given cycle. In contrast, in districts with the lowest risk associated with pandemic dynamics (“District 1”), the vaccination program was not initiated in any cycle. The result is the number of people who had contact with the virus in the analyzed districts:

- “District 1”—329,623 people
- “District 10”—232,946 people

It should be noted that in “District 1”, despite the total lack of vaccinations, only 11% of inhabitants got sick, and in “District 10”, with an active vaccination program, 20% of inhabitants got sick. The above data confirm that for PDPA, the main decisive factor in choosing which precinct to start the vaccination program in is the growth dynamics of infected people. The decrease of these dynamics can be obtained in two ways: by vaccination or by increasing the number of people who have already been in contact with the virus—the recovered persons.

The conclusions of PDPA, which, as already mentioned, is a more general solution of the vaccine distribution problem dedicated both to territorial units and to any other social groups, were the basis for the development of PDPAHIT. PDPAHIT is designed for territorial units where it is possible to achieve herd immunity. For the validation of the model, the threshold of collective immunity was assumed at the level of 75%. As in the previous case, the results for “District 1” and “District 10” will be used to discuss the results of PDPAHIT. The results for “District 10” are shown in Table 8, which includes the number of people vaccinated with each type of vaccine in the subsequent analyzed periods. In contrast, for “District 1” the model again did not allocate vaccines in any analyzed cycle. Analogously to PDPA, in PDPAHIT the distribution started from districts with the highest probability of infection and the largest populations while allowing the pandemic to develop in districts with smaller populations (“District 1”). The difference, however, is the cessation of vaccination in a given district once herd immunity is achieved. For example, for the presented “District 10”, this threshold is 886,150 vaccinated individuals. For the aforementioned district, the model achieves this result already in the first cycle, which allows for the termination of vaccination in this region and the initiation of vaccination in the next districts, according to the rules analogous to PDPA.

**Figure 1:**
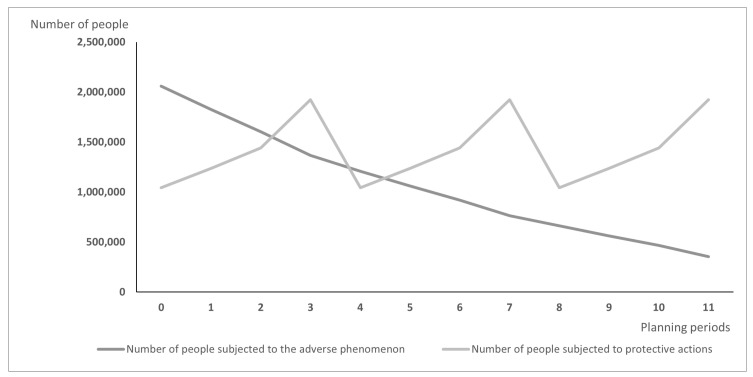
The results obtained using the PDPA model for the entire population.

**Figure 2:**
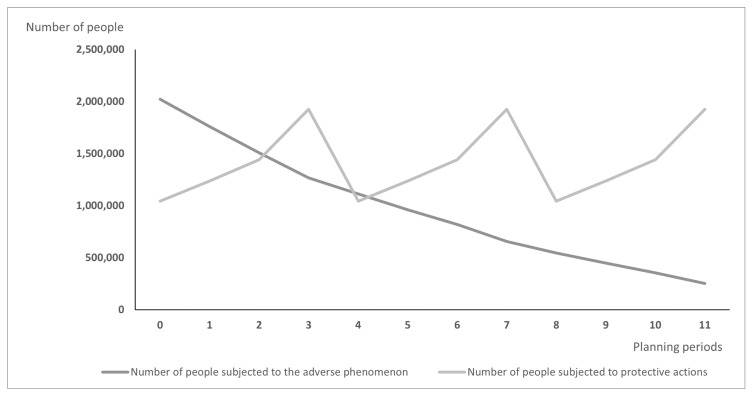
The results obtained using the PDPAHIT model for the entire population.

Differences between models may lead over time to the situation illustrated in Figure 1 and Figure 2: Figure 1 presents the hypothetic results when vaccines are distributed according to the PDPA model, while Figure 2 shows the number of exposed people and vaccinated people calculated using the PDPAHIT model. Although the results obtained in both problems may seem similar, it is worth emphasizing that the number of people subjected to the adverse phenomenon in the second experiment is smaller beginning with the first planning period. Both models allocate all available doses of protective actions (i.e., vaccines) in every planning period over the entire planning horizon. As the supply of vaccines was identical in both experiments, the plot of variable $x_{vrt}$ is the same in both figures. A strong assumption adopted in our research was the willingness of the whole social group to take the vaccine, which is reasonable when the protective action is addressed firstly to the most exposed groups, e.g., to health care workers just after the vaccine is approved. The plots of variable $I_{rt}$ are different; using the PDPAHIT model (Figure 2) we obtained in every planning period a smaller number of people exposed to the adverse phenomenon compared to the PDPA. It results from the relationships described in the previous paragraph, especially obtaining the herd immune threshold in social groups (i.e., districts).

## 4. Discussion

The optimization problems described in this paper focus on the issue of vaccine distribution during the first period of a pandemic when individual decision-making units face vaccine shortages. PDPA, which does not take herd immunity into account, can be used for social groups and territorial units. PDPAHIT, which takes achieving herd immunity into account, is dedicated to territorial units. The essence of linear programming models for the described problems is a strict definition of acceptable solutions to the problem of vaccine distribution, and then choosing from among these solutions the best one concerning the assumed optimization criterion—minimization of the number of infections. The results presented in Section 3 are an optimal solution, which means that with the adopted assumptions. There is no better method of vaccine distribution to minimize the number of infections. Both presented MIP models are based on the correlations described in Section 2 and are based on certain simplifications.

One such debatable factor is the failure to account for the time lag between the moment of vaccination and the attainment of manufacturer-defined immunity. It is important to note that detailed knowledge of vaccine performance is required to account for such a parameter, which may not be possible in the situation at hand—an initial vaccination program to combat a global pandemic. For example, in the case of the COVID-19 pandemic, even after one year of its duration, such information is inconclusive for all commercially available vaccines. On the other hand, including such a parameter as a variable defined by the decision-making unit seems reasonable. Another issue related to the above problem is vaccines taken in multiple doses and the resulting change in the probability of infection or severe disease. According to the authors, including this factor in the model as a defined vector of probabilities for particular vaccines is not a problem. The problem is, as above, the availability of such detailed information during the first months of vaccination.

Another issue is the probability $p_{r}^{R}$/$p_{v}^{V}$ used in the model, understood as the probability of infection or occurrence of a severe form of the disease. Currently, the decision-making unit must choose whether it is interested in minimizing the number of infections or reducing the incidence of a severe form of the disease and run the model with appropriately prepared data. According to the authors, the target model should account for both probabilities and the relationships between them. In such an approach, the target function can always aim to minimize the probability of death. For example, for the COVID-19 pandemic, the probability of infection depends mainly on contact with a sick person. Factors such as the population size in a given territory or occupation come into play here. The severity of the disease depends on the age of the infected person. It should be noted, of course, that PDPA can indicate the optimal distribution of vaccines for age groups with an assumed probability of severe disease or death.

Another problematic issue is assuming a constant probability of infection in the analyzed territorial units or occupational groups. As already noted by the authors, such a simplification seems reasonable in the absence of access to detailed data. However, the emergence of the COVID-19 pandemic has initiated several studies and works on modeling its development over time. Some of these studies are referenced in Section 1. In turn, the relationship between infectivity and the vaccination program was described by Higgins et al. [62]. Therefore, according to the authors, it seems reasonable that the target model should be based on a vector of infection probabilities. It will enable the combining of the optimization model with the prognostic models in the future. Such a tool will allow for a much better reflection in the model of the developing pandemic dynamics.

In summary, models aimed at scheduling a vaccination program during a pandemic of an unknown disease will always face a shortage of detailed information. Therefore, according to the authors, the simplified approach presented here can support decision-makers with minimal data requirements.

## 5. Conclusions

During emergencies of such a magnitude as a global pandemic, the ability to make fast yet optimal management decisions with limited access to information cannot be overestimated. Therefore, optimization models to support decisions on specific aspects of pandemic prevention and control appear to be necessary. The models presented in this work support the decisions regarding the initial distribution of a limited number of vaccines. Two MIP models are proposed to address this problem: (1) PDPA—not taking herd immunity into account, dedicated to social groups; and (2) PDPAHIT—taking into account herd immunity, dedicated to territorial units. Both approaches allow for the determination of the optimal vaccination schedule under the described conditions. As such, they are based on a minimal amount of data, which according to the authors (as shown in the discussion section), is not so much a drawback of this approach as a necessary compromise between the need for quick action and limited access to data (new viruses, new vaccines, etc.).

The models presented in the paper are theoretical and refer to the situation when the supply of vaccines (or any other protective actions) is not sufficient to cover the whole population. Throughout the paper, we intentionally refer to the distribution of protective actions, and we just treat vaccines as an example of such measures. The size of population (e.g., excluding people who are unwilling to take the protective action) and its stratification is the input to the model and is out of the scope of this research. In such circumstances, our models can be included in the decision support system to justify the decision of how to distribute the available number of protections (e.g., vaccine doses) so that the best possible protection can be obtained. Therefore, the problem we formulate and the model we choose is of real importance. Due to the simplifications listed in the manuscript, we indicate possible shortages of solutions (what is common for OR problems) we generate an optimal vaccination schedule, which is the ideal one under all adopted assumptions, and in real life can serve as the reference or the starting point for developing a schedule to be implemented in practice.

The next step in the further development of the described problems, according to the authors, is to prepare them to operate in a situation of greater access to data. Ultimately, such models should interact with other models predicting the development of a pandemic or analyzing the effects of particular vaccines on the organism. Such a holistic approach allows for changing the optimal model solution with increasing access to data during pandemic development.

## Figures and Tables

**Table 1:** The main factors underlying severe COVID-19 infection [10].

Age Range [Years]	Cases	Hospitalization	Death
0–4	<1x	2x	2x
5–17	Reference group	Reference group	Reference group
18–29	2x	6x	10x
30–39	2x	10x	45x
40–49	2x	15x	130x
50–64	2x	25x	440x
65–74	1x	40x	1300x
75–84	1x	65x	3200x
85+	2x	95x	8700x

**Table 2:** Characteristics of vaccines [11,12,13,14].

Characteristics	Vaccines			
	Pfizer	Moderna	AstraZeneca	JohnsonAndJohnson
Number of doses	2	2	2	1
Vaccine efficacy against COVID-19	0.950	0.941	0.595	0.669
Vaccine efficacy against severe COVID-19	no data	1	1	0.854

**Table 3:** Notation.

**Sets**		
T	=	the set of identical consecutive planning periods;
V	=	the set of available protective measures (e.g., different preparations) to prevent an adverse phenomenon (e.g., death, disease);
R	=	the set of homogeneous social groups defined based on a selected criterion (e.g., age, occupation, place of residence);
**Parameters**		
*M*	–	a sufficiently large constant;
$d_{r}$	–	the size of the group *r*;
$p_{r}^{R}$	–	the probability of occurrence of an adverse phenomenon in a person in a given group *r* not covered by protective measures;
$p_{v}^{V}$	–	the probability of occurrence of an adverse phenomenon in an individual covered by protective action *v*;
$b_{vt}$	–	maximum available number of units of protective action *v* in period *t*;
$C_{t}$	–	maximum available number of units of all protective actions in period *t*;
*f*	–	the percentage of applied protective actions ensuring collective immunity of the community to the adverse phenomenon;
**Decision Variables**		
$x_{rvt}$	–	the number of individuals subjected to the protective action *v* in group *r* in period *t*;
$I_{rt}$	–	the expected value of the number of individuals in group *r* subject to the adverse phenomenon in period *t*;
$y_{rt}$	=	1 if group *r* achieved herd protection in period *t*, else 0

**Table 4:** Assumed territorial division.

Name	Population Size	pR
District 1	2,901,225	0.01
District 2	2,077,775	0.06
District 3	2,117,619	0.05
District 4	1,014,548	0.08
District 5	2,466,322	0.03
District 6	3,400,577	0.03
District 7	5,403,412	0.07
District 8	986,506	0.04
District 9	2,129,015	0.09
District 10	1,181,533	0.09
District 11	2,333,523	0.07
District 12	4,533,565	0.04
District 13	1,241,546	0.06
District 14	1,428,983	0.09
District 15	3,493,969	0.06
District 16	1,701,030	0.09

**Table 5:** Assumed vaccine parameters.

Name	pR
Vaccine 1	0.950
Vaccine 2	0.941
Vaccine 3	0.595
Vaccine 4	0.669

**Table 6:** Assumed schedule of vaccine delivery [the number of people who can be vaccinated].

Period	Vaccine 1	Vaccine 2	Vaccine 3	Vaccine 4
0	873,000	0	172,000	0
1	873,000	204,000	161,000	0
2	873,000	0	268,000	300,000
3	873,000	287,000	765,000	0
4	873,000	0	172,000	0
5	873,000	204,000	161,000	0
6	873,000	0	268,000	300,000
7	873,000	287,000	765,000	0
8	873,000	0	172,000	0
9	873,000	204,000	161,000	0
10	873,000	0	268,000	300,000
11	873,000	287,000	765,000	0

**Table 7:** Model PDPA—District 10.

Period	Vaccine 1	Vaccine 2	Vaccine 3	Vaccine 4
0	172,000	0	0	0
1	0	0	204,000	0
2	0	0	0	0
3	92,911	0	0	630,527
4	0	0	0	0
5	0	0	0	0
6	0	0	0	0
7	0	0	0	0
8	0	0	0	0
9	0	0	0	0
10	0	0	0	0
11	0	0	0	0

**Table 8:** Model PDPAHIT—District 10.

Period	Vaccine 1	Vaccine 2	Vaccine 3	Vaccine 4
0	172,000	0	0	714,150
1	0	0	0	0
2	0	0	0	0
3	0	0	0	0
4	0	0	0	0
5	0	0	0	0
6	0	0	0	0
7	0	0	0	0
8	0	0	0	0
9	0	0	0	0
10	0	0	0	0
11	0	0	0	0

## Data Availability

Not applicable.

## Abbreviations

The following abbreviations are used in this manuscript:

DMU	Decision Making Unit
DSS	Decision Support System
MIP	Mix-Integer Programming
NPI	Non-Pharmaceutical Interventions
PDPA	Problem of Distributing Protective Actions
PDPAHIT	Problem of Distributing Protective Actions with a Herd Immunity Threshold
